# A systematic review and meta-analysis on different stem fixation methods of radial head prostheses during long-term follow-up

**DOI:** 10.3389/fbioe.2022.1041531

**Published:** 2022-11-01

**Authors:** Guang Yang, Shangzhe Li, Hailong Zhang, Yi Lu

**Affiliations:** Department of Sports Medicine, Beijing Ji Shui Tan Hospital, School of Medicine, Peking University, Beijing, China

**Keywords:** radial head, arthroplasty, replacement, prostheses, fixation

## Abstract

**Background:** Radial head arthroplasty (RHA) is typically performed for non-reconstructible radial head fractures with or without valgus stability. The fixation methods can be divided into cemented rigid fixation, such as screw fixation, and uncemented micromovement fixation, including smooth stem, press-fit, expanded device, in-growth stem, and grit-blasted stem fixations. Different fixation methods may impact long-term clinical outcomes and cause complications. This study aimed to compare the long-term follow-up outcomes of cemented and uncemented radial head prostheses.

**Methods:** A computerized literature search was performed in the PubMed/MEDLINE, Embase, Cochrane Library, and Web of Science databases for studies on radial head prostheses, replacement, and arthroplasty published from inception to April 2022. The prostheses fixation method was divided into cemented and uncemented fixation groups. The outcomes of interest included the participant characteristics, prostheses types, clinical outcomes, reoperation rates, and complication rates during long-term follow-up.

**Results:** A total of 57 studies involving 2050 patients who underwent RHA were included in our analysis. Cemented fixation was used in 23 of these studies, uncemented fixation in 35 studies, and both cemented and uncemented fixations in one study. Both fixation groups showed significantly improved clinical outcomes after treatment. In particular, both the reoperation and complication rates were lower in the uncemented fixation group (12% and 22%, respectively) than that in the cemented fixation group (20% and 29%, respectively). Among the studies, uncemented monopolar fixation had the lowest reoperation rate (14%), while cemented monopolar fixation had the highest reoperation rate (36%). Regarding complication rates, uncemented bipolar fixation yielded the lowest rate (12%), while cemented bipolar fixation yielded the highest rate (34%). The range of motion and clinical outcome scores were good in both groups.

**Conclusion:** Uncemented radial head prostheses had lower reoperation and complication rates than cemented prostheses. In particular, uncemented monopolar prostheses may yield the lowest reoperation rate, while uncemented bipolar prostheses may yield the lowest overall complication rate.

## Introduction

Radial head fractures account for approximately 33% of elbow fractures and 1.7%–5.4% of all fractures (Mason ML, 1954; Kaas L et al., 2010). When dislocation or comminuted radial head fractures (modified Mason types III and IV) occur, it is crucial to restore radial head function because of its essential role in maintaining elbow stabilization (Morrey et al., 1991). Currently, radial head arthroplasty (RHA) is universally accepted for the treatment of comminuted radial head fractures. Sershon et al. reported that approximately 85% of patients achieved good-to-excellent outcomes after primary RHA (Sershon et al., 2018). Li et al. performed a systematic review and meta-analysis and concluded that the application of RHA led to better range of motion (ROM) and lower complication rates (Li and Chen, 2014). However, complications after RHA cannot be avoided, with varying reoperation rates ranging from 0% to 45% (Laumonerie et al., 2017). Among these complications, painful loosening is the primary reason for radial head fixation reoperation (Van Riet et al., 2010; O’Driscoll and Herald, 2012; Duckworth et al., 2014; Delclaux et al., 2015; Neuhaus et al., 2015; Kachooei et al., 2016); its cause is speculated to be related to the manner the prostheses stem is fixed.

Radial head prostheses fixation can be divided according to the stem fixation method: cemented and uncemented. Cemented fixation is defined as firm fixation of a prostheses in the medulla without micromotion. Meanwhile, uncemented fixation is described as fixation of a prostheses in the medullary region with micromotion owing to a 1–2 mm space existing between the prostheses stem and medullary cavity. Previous studies have reported that cemented prostheses can achieve good stability, with a lower loosening rate (Acevedo et al., 2014), while uncemented micromotion prostheses can avoid the oversizing effect, alleviate radial head impingement, and have fewer complications (Chanlalit et al., 2012; Laflamme et al., 2017). Nevertheless, there is still controversy regarding which type of prostheses has better performance.

The purpose of this study was to compare between cemented and uncemented radial head prostheses during long-term follow-up. We hypothesized that uncemented prostheses have lower complication and reoperation rates than cemented prostheses.

## Methods

### Search strategy

The study was performed in accordance with the Preferred Reporting Items for Systematic Reviews and Meta-Analyses (PRISMA) guidelines. The PubMed/MEDLINE, Embase, Cochrane Library, and Web of Science databases were searched from inception to April 2022 using the following search terms: radial head, radial head arthroplasty, prostheses, unipolar, monopolar, bipolar, cemented, uncemented, and not reviewed. Randomized controlled trials, retrospective cohort studies, and case series were included, and the average follow-up period was >24 months. The prostheses fixation methods were divided into cemented and uncemented fixation groups. Letters, comments, editorials, case reports, proceedings, and personal communications as well as studies in which the characteristics of elbow injury involved active infection, previous treatment failure or bilateral treatment were excluded. The list of potential references was reviewed, and data were extracted by two independent reviewers; a third reviewer was consulted to resolve any uncertainties regarding eligibility.

### Data extraction and quality assessment

The following data were extracted from the studies that met the inclusion criteria: name of the first author, year of publication, design of the study, number of participants in each group, age and sex of the participants, implants used for RHA, complications, length of follow-up, and major clinical functional outcomes.

The quality of the included studies was evaluated using the modified 18-item Delphi checklist.

### Outcome measures

The primary outcomes were the rates of reoperation and overall complications. The secondary outcomes were the average range of clinical functional outcomes, including the mean Mayo Elbow Performance Score (MEPS) and Disabilities of the Arm, Shoulder, and Hand (DASH), visual analog scale (VAS), The American Shoulder and Elbow Surgeons Elbow Questionnaire (ASES), and Broberg–Morrey scores, and the average ROM (flexion minus extension). For subgroup analysis, we would investigate the effect of prostheses polarity on the rates of reoperation and complications.

### Statistical analysis

Event rates with 95% confidence intervals (Cis) were calculated for dichotomous outcomes and means with 95% Cis for continuous outcomes. Heterogeneity among the studies was assessed using Cochran’s Q and I^2^ statistics. The I^2^ statistic indicates the percentage of the observed between-study variability caused by heterogeneity. Heterogeneity was evaluated based on the I^2^ statistic as follows: 0%–24%, no heterogeneity; 25%–49%, moderate heterogeneity; 50%–74%, large heterogeneity; and 75%–100%, extreme heterogeneity. When the I^2^ statistic (>50%) indicated heterogeneity between the studies, the random-effects model (DerSimonian–Laird method) was used. Otherwise, the fixed-effects model was utilized (Mantel–Haenszel method). Pooled effects were calculated, and a two-sided *p* value of <0.05 was considered to indicate statistical significance. All statistical analyses were performed using the RevMan software version 5.3 (The Cochrane Collaboration, Oxford, United Kingdom).

## Result

**FIGURE. 1:**
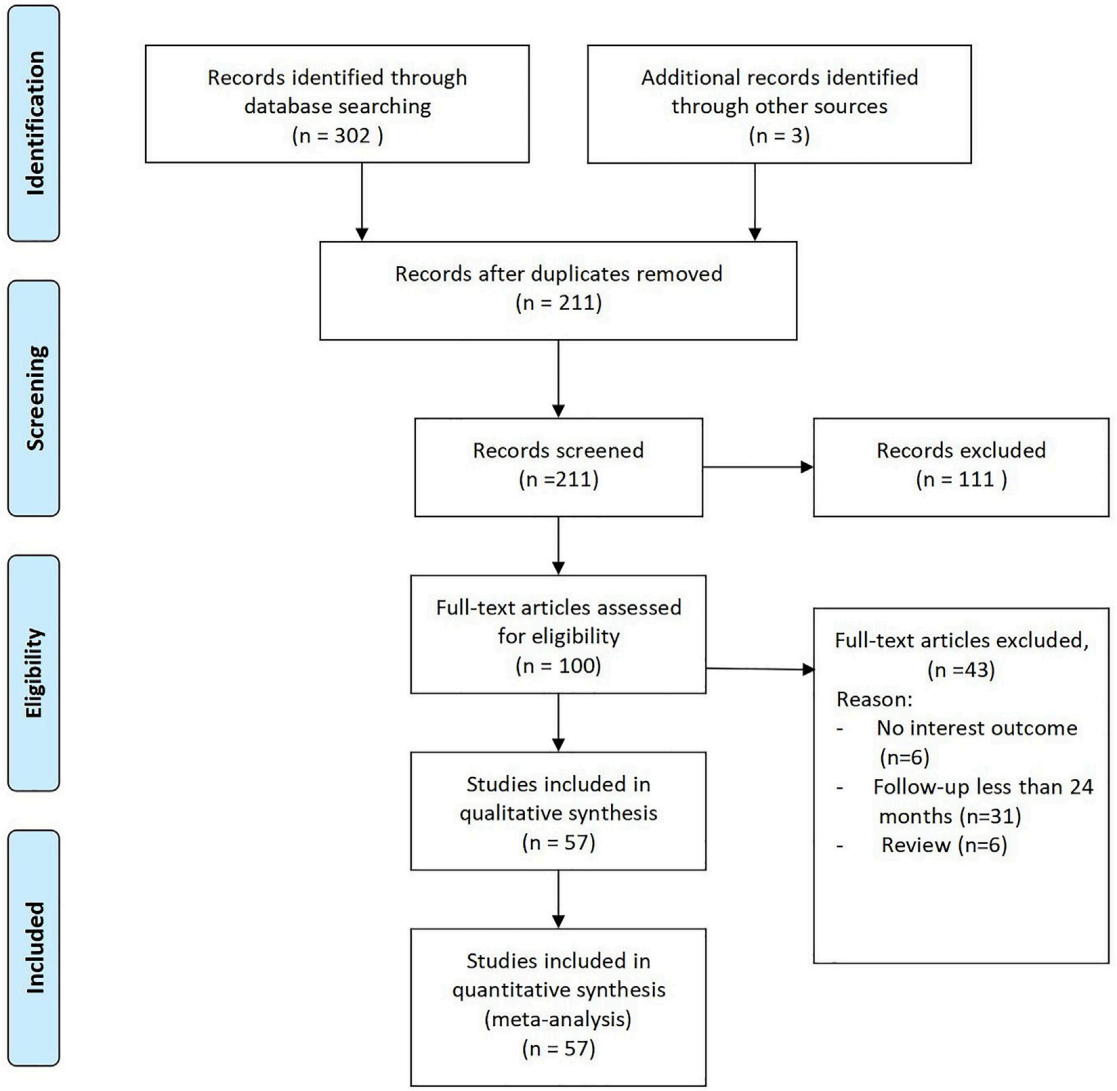
PRISMA 2009 flow diagram.

Of the 211 studies initially identified, 111 were excluded after preliminary screening of the abstracts and titles because they were not relevant (Figure 1). The remaining 100 articles underwent full-text review, and 42 were excluded for not reporting the outcomes of interest, not applying RHA, and not mentioning whether the periprosthetic fixation method was cemented fixation.

57 studies were finally included in the analysis, with a total of 2050 patients who received RHA implants: 618 patients received cemented implants, and 1,432 patients received uncemented implants. Two studies were prospective studies; eighteen, retrospective studies; and thirty-seven, case series (Table 1). Across all studies, the mean age in the cemented and uncemented fixation groups was 47.4 and 49.5 years, respectively; the number of the male patients in the cemented and uncemented fixation groups was 320 and 601, respectively, while that of the female patients was 214 and 631 in the studies that mentioned related item, respectively. In general, the average of follow-up in cemented group was 65.6 months, and that in uncemented group was 60.0 months. The detail is shown in Table 1 and 2.

**TABLE. 1:** Baseline demographics of the selected studies for cemented fixation.

Author (year)	Type of studies	Patients	Average age	Gender (male)	Dominant	Average follow-up (month)	Indication of surgery	Implant of radial head arthroplasty	Polarity (monopolar VS. bipolar)	Clinical outcomes	Rang of motion (flexion minus extension)	Reoperation	Complications	QA
Luo2022	Case series	17	35	15	13	103	stiffness by trauma	Tornier	bipolar	MEPI: 95 ± 6; DASH: 8 ± 5; VAS: 0.2 ± 0.5	ROM:113 ± 19°		ulnar nerve symptoms n = 1	13
Songy2021	Retrospective Cohort	29				78	acute, nonreconstructible radial head fractures	Evolve n = 60; SBI/Avanta/Stryker n = 21; Anatomic n = 33	monopolar			4	stiffness:pain:Transverse instability/pain n = 1:2:1	15
Pehlivanoglu2021	Retrospective Cohort	26	48.85	15		132.2	acute and comminuted radial head fractures	OrtoPro	monopolar	VAS:0.8; MEPS:91.5; DASH:6.3; SF-36:55.5	ROM:117.5°	1	periprosthetic stem lucency and pain n = 1	11
Montbarbon2021	Case series	16	52.5	9	8	144		Tornier	bipolar	Quick-Dash:23.01 ± 7.8	ROM:132 ± 11°	3	recurrent dislocation n = 1; or chronic pain n = 1	17
Marcheix2021	Case series	41	59	14		86.9	acute elbow trauma	Judet-type RH prostheses with floating cup	bipolar	VAS:0.78, MEPI:88.7, DASH18.7	ROM:13–139.3°	5	regional pain:nerve palsy n = 4:1	15
Laun2019	Case series	37	49.9	21	28	67.2	radial head fracture Mason III	Judet’s bipolar prostheses	bipolar	VAS: 1.2; DASH:18.6; Broberg and Morrey score:86.5; MEPS: 90.3	ROM: 12.5–131.7°	1		17
Kiechle2019	Retrospective cohort study	48	45			46	fracture associated with instability of the elbow	MoPyC; Tornier	monopolar	VAS:3.3; MEPS:70 Broberg and Morrey score:63 DASH:34	ROM: 20–118°		subluxation:loosening:ossifications:nerve affection:instability n = 1:1:1:1:1	16
Laumonerie2018	Retrospective cohort study	65	52.46	44		76.78	nonreconstructable fracture	GUEPAR n = 30; Evolutive n = 20; rHead STANDARD n = 6; rHead RECON prostheses n = 9	bipolar n = 59; monopolar n = 6	MEPS:87.4; DASH:16.57	ROM: 11–133.6°	14	painful loosening n = 14	14
Jungbluth2018	Case series	46	57.7	19	35	65.3	Monteggia-like lesion	Tornier	bipolar	VAS:1.0; Morrey and broberg score: 86.6; DASH:15.1	ROM: 8–133.2°	6		13
Cui2018	Retrospective cohort study	9	31	5	3	29	Terrible triad injury of the elbow	Edina	bipolar	MEPS:94	ROM: 5–126°			9
Laumonerie2017	Retrospective cohort study	77	52	54	42	74	nonreconstructable fracture	Guepar n = 36; Evolutive n = 24; rHead RECON; SBI/Stryker n = 10; rHead Standard n = 7	bipolar n = 70; monopolar n = 7	MEPS:88.8; DASH:15.5	ROM:9.3–135.3°	30	painful loosening n = 29; nerve palsy n = 12	12
Lopiz2016	Retrospective cohort study	14	54	6	6	42	radial head fractures	MoPyC	bipolar	DASH:24.8 MEPI:78.9	ROM:85.5°	4	stiffness n = 3; fracture n = 1; neurological injuries n = 2	15
Heijink2016	Case series	25	55	7	4	50	radial head fractures	Tornier	bipolar	MEPS:89.6	ROM:6–135°	1	neuropraxia n = 2; nerve paresthesia n = 2; stiffness n = 1	14
Alllavena2014	Case series	22	44	15	10	50	radial head fractures	The Guepar® radial head prostheses	bipolar	MEPS:79	ROM:100	4	postero-lateral subluxation n = 6; nerve dysfunction n = 5	13
Liu2013	Case series	8	31.7	7		26	elbow stiffness	Tornier, Edina, MN, United States	bipolar	MEPS:92.5	ROM:7.5–120.6°			17
Leigh2012	Retrospective cohort study	11	45.5	6		40.7	terrible triad injuries	Avante or Evolve	monopolar	DASH:10.83 ASES:89	ROM:5–135°	4	stiffness n = 2	16
Celli2010	Case series	16	46.1	11	9	41.1	radial head fractures	Tornier, Edina	bipolar	VAS:1.38; MEPS:89.4; DASH:11.4	ROM:117°		ankylosis n = 2 synostosis n = 2	13
Burkhart2010	Case series	17	44.1	14		106	radial head fractures	Judet’s bipolar radial head prostheses	bipolar	MEPS:90.83 DASH:9.8	ROM:21–124°		dislocation n = 2	14
Lim2008	Case series	7		2	4	29.7	radial head fractures	Howmedica	monopolar	VAS:1.8; Broberg and Morrey score:78.4; DASH:13.61; ASES:92.5	ROM:100°		neuropathy n = 1; loosening n = 4	14
Popovic2007	Case series	51	51	32	41	100.8	radial head fractures	Tornier	bipolar	MEPS:83	ROM:14–130°		regional pain syndrome n = 1; nerve palsy n = 5; subluxation n = 1	8
Dotzis2006	Case series	14	44.8	10	9	63.6	radial head fractures	Tornier	bipolar	DASH:23.9	ROM:14–140°		complex regional pain syndrome n = 1	16
Brinkman2005	Case series	11	43	8	6	24	treated previously with ORIF	Judet CRF II	bipolar		ROM:14.5–135°	2		15
Popovic2000	Case series	11	52.7	6	7	32	radial head fractures associated with elbow dislocation	Tornier	bipolar		ROM:14.5–130°			11

**TABLE 2:** Baseline demographics of the selected studies for uncemented fixation.

Author (year)	Type of studies	Patients	Average age	Gender (male)	Dominant	Average follow-up (month)	Identification of surgery	Implant of radial head arthroplasty	Polarity (monopolar VS. bipolar)	Clinical outcomes	Rang of motion (flexion minus extension)	Reoperation	Complications	QA
Gramlich2021	Retrospective comparative treatment study	66	48	41		42.2	nonreconstructible radial head fractures	SBI rHead n = 31; Tornier MoPyC n = 35	bipolar n = 31; monopolar n = 35		ROM:17.7–127.5°	13	painful loosening n = 6; joint stiffness n = 3	13
Raven2020	Retrospective cohort study	86	54	37	46	87.6	nonreconstructible radial head fractures	Evolve n = 75; MoPyC, Tornier n = 11	monopolar	MEPI:79.4; DASH: 24.5	ROM:16.5–128.2°	4	painful loosening n = 5; nerve syndrome n = 3	14
Songy2021	Retrospective cohort study	85				78	persistent symptoms previously treated radial head fractures	Evolve n = 60; SBI/Avanta/Stryker n = 21; Anatomic n = 33	monopolar			10	stiffness n = 1; pain n = 7	12
Mukka2020	Retrospective cohort study	14	45	7	7	72	comminuted radial head fractures	Explor n = 14	monopolar	VAS:2; DASH:26	ROM:17–125°			11
Claessen2020	Case series	24	48	8		27	nonreconstructible radial head fracture associated with elbow instability	Tornier						15
Chen2020	Retrospective cohort study	33	44.76	20	18	108.36	comminuted radial head fractures	Evolve	monopolar	MEPS:84; DASH:10.8	ROM:126.8°	1	stiffness n = 1	15
Carbonell-Escobar2020	Case series	62	54		32	62.4	nonreconstructible radial head fracture associated with elbow instability	Evolve n = 27; Anatomic Radial Head n = 35	monopolar	MEPS:83	ROM:10–125°		neurologic symptoms n = 10	16
Baek2020	Case series	24	49.8	13		58.9	complex radial head fractures with associated injuries	EVOLVE n = 10; Acumed n = 7; Zimmer-Biomet n = 5; Tornier n = 2	monopolar	VAS:0.6 MEPS:88.7; DASH: 19.4	ROM:4.7–132.7°	1	stiffness n = 2; ulnar neuropathy n = 2	14
Jung2019	Retrospective cohort study	57	49	31	22	100.8	nonreconstructible radial head fracture	Evolve	monopolar	MEPS:74 ± 22; DASH:31 ± 25; VAS:2.1 ± 2.5	ROM:102°	12	loosening n = 7; instability n = 1; nerve syndrome n = 4; stiffness n = 1; pain syndrome n = 1	13
Gramlich2019	Retrospective cohort study	66	48	41		42	acute radial head fracture	rHead, SBI n = 31; MoPyc, Tornier n = 35	bipolar n = 31; monopolar n = 35			13	painful loosening n = 17; stiffness n = 3	12
Cristofaro2019	Case series	119	50	56		132			monopolar	DASH:13		30	painful loosening n = 6; stiffness n = 12	13
Ricon2018	Case series	18	48	13		79.8		MoPyC, Tornier	monopolar	MEPS:89.5	ROM:15–127°			15
Tarallo2017	Case series	31	49	26	26	30	radial head fractures	Acumed		MEPS:91.2	ROM:112°			14
Strelzow2017	Prospective study	148	55	43	66	56.4	radial head fractures	Evolve	monopolar	DASH:17.55	ROM:14–135°	5		12
Laflamme2017	Retrospective cohort study	57	50.2	28	27	75.6		The EVOLVE n = 32; The ExploR n = 48	monopolar	VAS:1.11; DASH:7.7; MEPI:96.5		2		11
Han2016	Case series	3	53.3	0	2	24.6	isolated radial head fracture	SBI		MEPS:95 DASH:7.5 ASES:94.7	ROM:6.6–140°			15
Gauci2016	Case series	65	52	30	36	46	radial head fractures	MoPyC, Tornier	monopolar	VAS:1; MEPS:96	ROM:9–136°	4		17
Yan2015	Retrospective cohort study	20	36.54	11		36	radial head fractures with terrible triad	Waldemar LINK GmbH & Co.	monopolar	MEPS:85.8	ROM:17–117°		stiffness n = 1; Secondary coronoid fragment displaced n = 1	7
Sarris2012	Case series	32	54	20	22	27		MoPyC, Tornier	monopolar		ROM:130°		stem neck dissociation n = 1	15
Rotini2012	Retrospective cohort study	30	44	19		24	radial head fractures with elbow stiffness or instability	SBI	monopolar n = 12; bipolar n = 19	MEPS:90		2		17
Ricon2012	Case series	28	54	11	15	32.6		MoPyC	bipolar	MEPS:92	ROM:15–120°		neuropathy n = 1	16
Flinkilla2012	Case series	42	56	16		53	acute unstable injury	Avanta Orthopedics n = 19; Acumed n = 23	monopolar	MEPS:86 DASH:23	ROM:20–135°		nerve palsy n = 1; stiffness n = 4	14
Lamas2011	Case series	47	51	18	32	48	nonreconstructable radial head fracture	Mopyc, Bioprofile-Tornier	bipolar	VAS:1	ROM:6–140°	3	dislocation n = 2; neurologic symptoms n = 2	16
Chen2011	Prospective randomised controlled trial	22	37			33.6	nonreconstructable radial head fracture	Evolve	monopolar	Broberg and Morrey:92.1			stiffness n = 3	15
Chien2010	Case series	13	37	10		38.3	radial head fractures	Evolve	monopolar	MEPS:86.9	ROM:6.2–126.5°	2	stiffness n = 2	15
Fehringer2009	Case series	16	55	9		32	comminuted radial head fractures	Evolve	monopolar					14
Shore2008	Case series	31	54		12	96	Chronic posttraumatic elbow disorders	Smith and Nephew Richards n = 22; Evolve n = 10	monopolar	MEPS:83			neuropathy n = 4; chronic regional pain syndrome n = 1	15
Anneluuk2008	Retrospective cohort study	31	47	22		24		EVOLVE n = 16; Swanson Titanium Radial Head Implant n = 1		MEPS:88; DASH:16.5	ROM:16–132°			13
Doornberg2007	Case series	27	52	13	7	48	radial head fractures	Evolve		MEPS:85 DASH:17 ASES:84	ROM:20–131°			17
Wretenberg2006	Case series	18	52	11		44.4	radial head fractures	Waldemar Link, GmbH & Co.		VAS:0.8	ROM:15–130°			12
Grewal2006	Case series	26	54	9	11	24.5	radial head fractures	Evolve	monopolar	MEPI:80.5 DASH:24.4	ROM:24.9–138.1°		neurologic n = 3	14
Ashwood2004	Case series	16	45	8	16	33.6	radial head fractures	Evolve	monopolar	MEPS:87 VAS:1.7	ROM:15–125°		regional sympathetic mediated pain n = 1; ulnar nerve neuropathy n = 3	15
Moro2001	Case series	24	54	11		39	radial head fractures	Smith and Nephew Richards	monopolar	MEPI:80; DASH:17	ROM:8–140°		regional sympathetic mediated pain n = 1; ulnar nerve neuropathy n = 1	16
Harrington2001	Case series	20	46	7	11	145.2	radial head fractures	Smith and Nephew Richards	monopolar	Broberg and Morrey score:88	ROM:17–120°	4	pain n = 4	9
Knight1993	Case series	31	57	12		54	comminuted radial head fracture	Silastic: Dow Corning	monopolar				ulnar nerve paraesthesia n = 2	12

### Quality assessment

The study quality was assessed using the modified 18-item Delphi checklist (maximal [i.e., best] score: 18). In general, the studies were of good quality, with most (55/59) studies having a score of >10. Two studies had a score of nine and other two studies had scores of 7 and 8, respectively.

### Cemented and uncemented fixation comparison

**FIGURE. 2:**
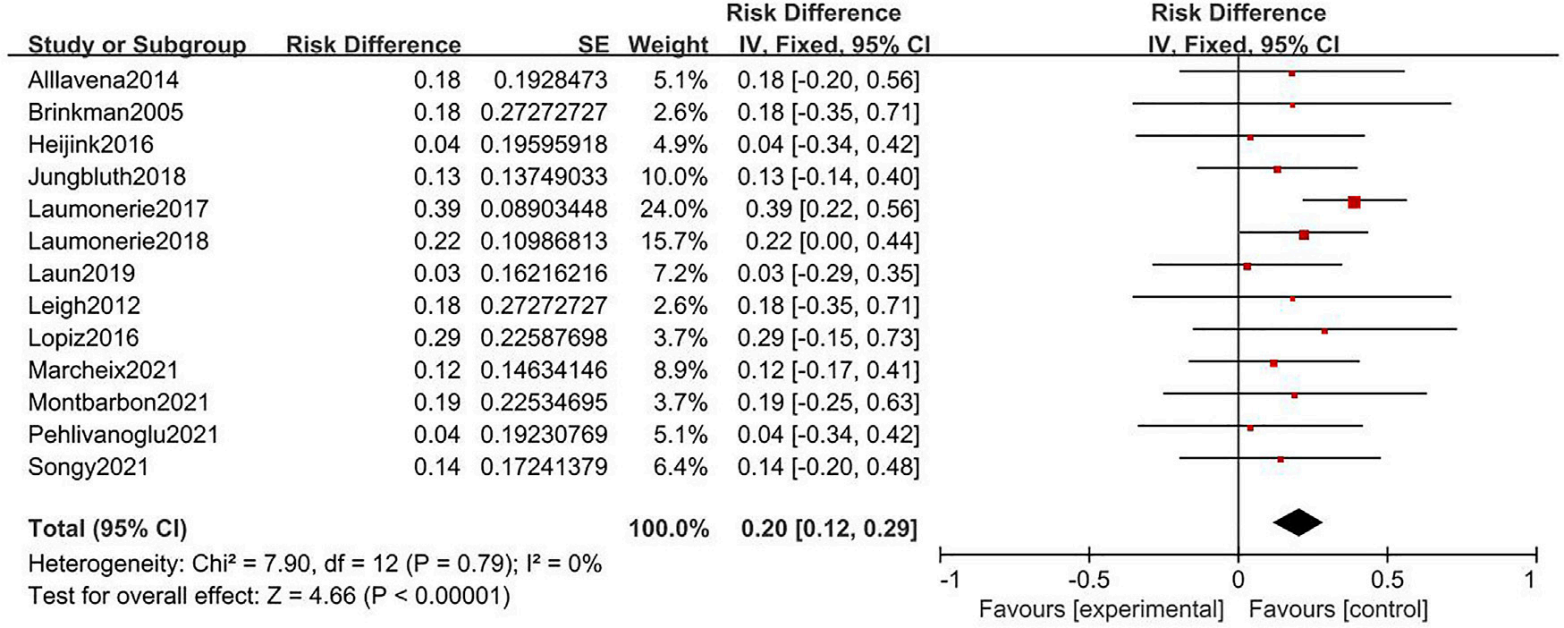
Forest plot for rate of reoperation of cemented fixation.

**FIGURE. 3:**
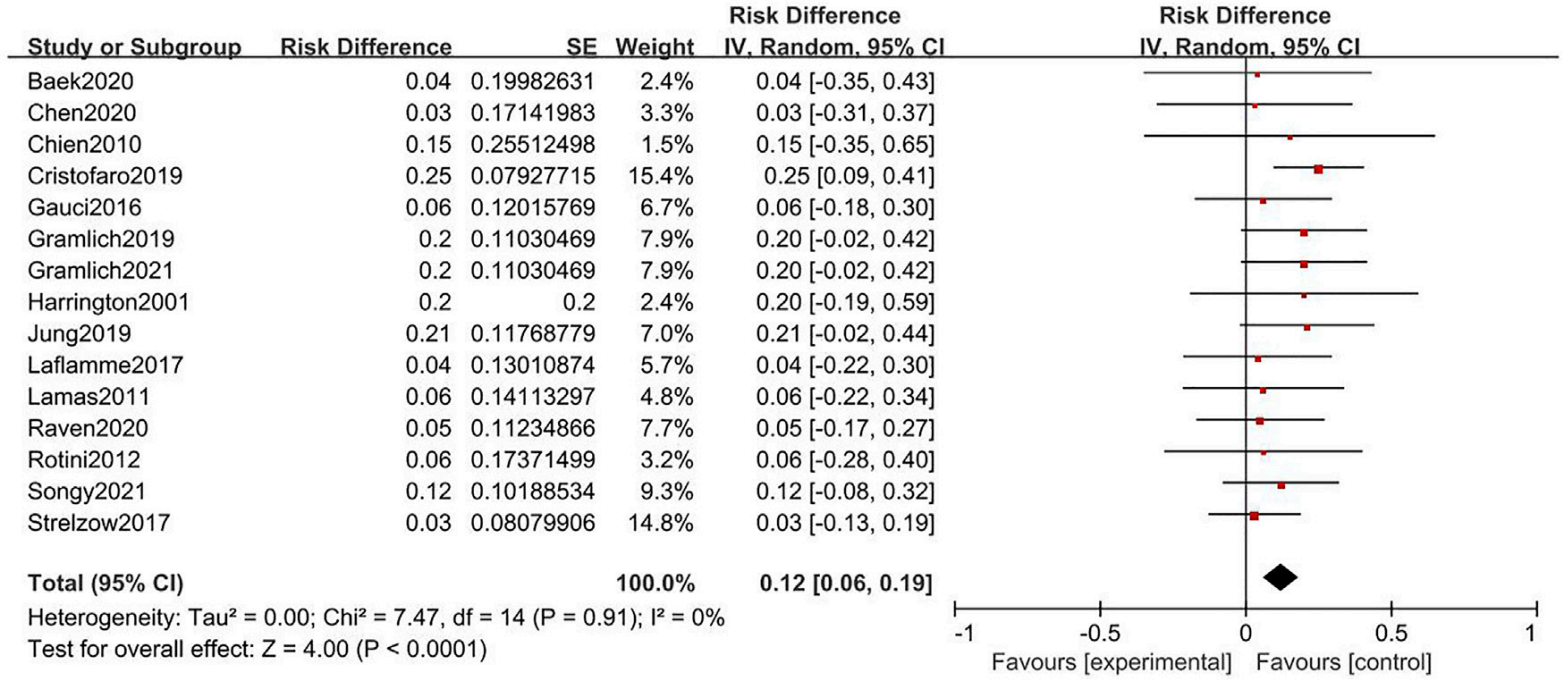
Forest plot for rate of reoperation of uncemented fixation.

**FIGURE. 4:**
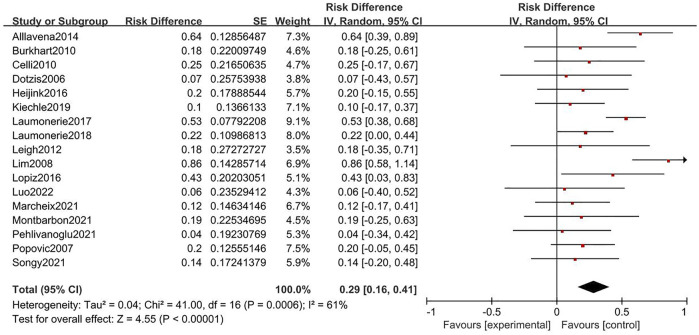
Forest plot for rate of complication of cemented fixation.

**FIGURE. 5:**
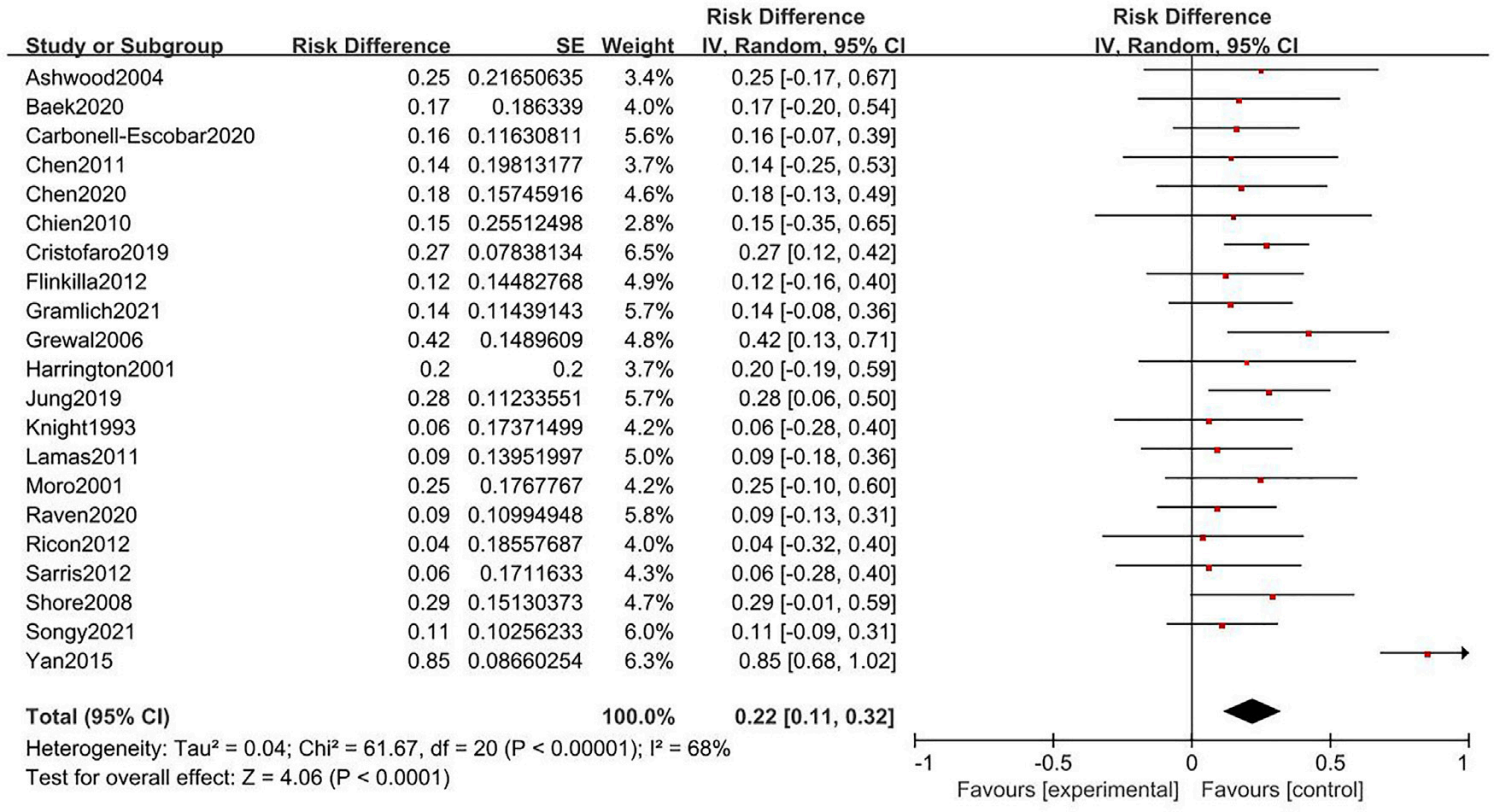
Forest plot for rate of complication of uncemented fixation.

Twenty-three and thirty-five studies were included in the analysis of cemented and uncemented fixations, respectively. No significant heterogeneity was found in the rate of cemented and uncemented reoperations (cemented: Q = 7.9, I^2^ = 0%; uncemented: Q = 7.47, I^2^ = 0%); therefore, the fixed-effects model was used. The rate of cemented reoperation ranged from 3% to 39%, while that of uncemented reoperation ranged from 2% to 25%, with pooled estimates of 20% and 12%, respectively (cemented: 95% CI: 0.12–0.29, uncemented: 95% CI: 0.06–0.19) (Figures 2, 3). Heterogeneity was observed in the overall complication rate (cemented: Q = 41; I^2^ = 61%, uncemented: Q = 61.67; I^2^ = 68%). Therefore, the random-effects model was used. The pooled estimate of overall complications in the cemented and uncemented fixation groups was 29% and 22%, respectively (cemented: 95% CI: 0.16–0.41, uncemented: 95% CI: 0.11–0.34) (Figures 4, 5). Moreover, the mean MEPS and DASH, VAS, ASES, and Broberg–Morrey scores were 70–95, 6.5–34, 0–3.3, 86–92.5, and 63–90.1 in the cemented fixation group and 74–96, 6.17–31, 0.6–2.9, 70–94.7, and 85.5–94.2 in the uncemented fixation group, respectively. The mean ranges of flexion, extension, pronation, and supination between the cemented and uncemented fixation groups were 118–140 vs 124–145, 5–21 vs 4.7–34, 43–87.5 vs. 47.9–85, and 57–88 vs. 35–85, respectively. Seventeen and twenty-two studies reported complications in the cemented and uncemented fixation groups, respectively. The following complications were found in the cemented fixation group: painful loosening (10.1%), nerve symptoms (6.5%), elbow stiffness (2%), dislocation (2%), and regional pain syndrome (1%). Meanwhile, the following complications were reported in the uncemented fixation group: painful loosening (4.3%), nerve symptoms (3.7%), elbow stiffness (3.5%), regional pain syndrome (1.6%), and dislocation (0.5%).

### Subgroup analysis

Eighteen studies used cemented bipolar prostheses; six, cemented monopolar prostheses; five, uncemented bipolar prostheses; and twenty-five, uncemented monopolar prostheses. In the meta-analysis, the lowest rate of reoperation was reported for uncemented monopolar fixation (14%, 95% CI: 0.08–0.20) and the lowest rate of complications for uncemented bipolar fixation (12%, 95% CI: 0.05–0.30). The highest rate of reoperation was reported for cemented monopolar fixation (36%, 95% CI: 0.15–0.56) and the highest rate of complications for cemented bipolar fixation (34%, 95% CI: 0.26–0.42) (Table 3). The range of clinical outcomes in cemented bipolar and monopolar was MEPS: 78.9–95 and 70–91.5, DASH: 8–23.9 and 6.3–34, VAS: 0.2–1.38 and 0.8–3.39, Broberg and Morrey: 86.5–86.6 and 63–78.4, while ASES score was only mentioned in two studies using cemented monopolar prostheses, it ranged from 89 to 92.5. And the range of that uncemented bipolar and monopolar was MEPS: 90–92 and 74–96.5, VAS: one and 0.6–2.1, while DASH and Broberg and Morrey reported in studies using uncemented monopolar were 7.7–31 and 88–92.1, respectively. For some of the more concerned complications, distribution of complications in cemented bipolar: nerve symptoms 0.05%, painful loosening 8%, elbow stiffness 2%, dislocation 2.9%, regional pain syndrome 2%, while that in studies using cemented monopolar: nerve symptoms 3.3%, painful loosening 33%, elbow stiffness 5%, dislocation 1.7%, regional pain syndrome 3.3%. Distribution of complications in uncemented bipolar: nerve symptoms 2%, painful loosening 17.5%, elbow stiffness 6%, dislocation 2%, while that in studies using uncemented monopolar: nerve symptoms 3.9%, painful loosening 3%, elbow stiffness 3.2%, dislocation 0.2%, regional pain syndrome 1.7%.

**TABLE 3:** Meta-analysis for secondary outcomes.

	Reoperation				Complications			
								
	Heterogeneity		Pooled results		Heterogeneity		Pooled results	
								
	Number of studies	I^2^	Effect size (95% CI)	p value	Number of studies	I^2^	Effect size (95% CI)	p value
								
Uncemented monopolar	16	0%	0.14(0.08,0.20)	<0.001	19	69%	0.24 (0.12,0.35)	<0.001
Cemented monopolar	4	0%	0.36(0.15,0.56)	<0.001	5	80%	0.32 (0.17,0.46)	<0.001
Uncemented bipolar	3	39%	0.20(0.02,0.37)	0.03	3	0%	0.12 (-0.05,0.30)	0.17
Cemented bipolar	12	34%	0.34(0.26,0.42)	<0.001	12	46%	0.34 (0.26,0.42)	<0.001

## Discussion

The main finding of this study is that uncemented radial head prostheses had lower reoperation and overall complication rates than cemented radial head protheses. In particular, uncemented monopolar prostheses yielded the lowest reoperation rate and uncemented bipolar prostheses, the lowest overall complication rate.

Multiple radial head prostheses fixation can be classified according to the stem fixation method: uncemented or cemented. For uncemented fixation, there is often a gap of 1–2 mm between the prosthetic stem and medullary cavity, allowing micromotion, which can lead to excessive stress that is appropriately dispersed. Muhm et al. conducted a survey on the outcome of uncemented prostheses during mid-term follow-up; they found a mean Broberg–Morrey score of 85.5 ± 12.2, indicating good results observed in the group (Muhm et al., 2011). For cemented stem fixation, the prostheses was rigidly fixed to the medulla using bone cement. Cement fixation, including firm fixation, may reduce early prostheses loosening, and smaller-diameter stems avoid iatrogenic fracture during surgery. Agyeman et al. performed a systematic review and meta-analysis and found no significant outcome between the cemented prostheses group and the loose smooth stem group, although the cemented prostheses group had a higher risk of complications and reoperation than the other group (Agyeman et al., 2019). Laumonerie et al. conducted a survey of prostheses implanted *via* cemented fixation and found a relatively high rate of painful loosening (approximately 21.5%), osteolysis (53.8%), and overstuffing (46.2%) (Laumonerie et al., 2018). Szmit et al. performed a biomechanical study and found that using cemented fixation would make the radial head prostheses less effective in distributing high contact stress and easier to get abraded (Szmit et al., 2019). Although Kachooei et al. observed the lowest rate of revision with cemented fixation in their systematic review and meta-analysis, they thought that the rate may be related to difficulties in removing the implant design (Kachooei et al., 2018). Regarding radiographic evaluation, Popovic et al. observed progressive radiographic loosening lines in 16 of 51 patients, and further 16 patients had loss of proximal bone support at the neck of the radius (Popovic et al., 2007). Similarly, Marcheix et al. found that 24% of elbows had radiographic loosening, and 54% of elbows developed lateral condyle demineralization (Marcheix et al., 2021). The results of the present study are comparable with those of previous studies. Herein, higher rates of reoperation and overall complications were found with cemented fixation than with uncemented fixation; in particular, the painful loosening rate was higher with cemented fixation than with uncemented fixation (10% vs 5.5%).

In addition, radial head prostheses were divided into monopolar and bipolar prostheses. Most monopolar prostheses are one-piece unipolar metallic devices with a closely connected junction between the radial head and the stem. Strelzow et al. conducted a survey on monopolar prostheses and found that the final mean PREE and Quick-DASH scores were 17 ± 3 and 14 ± 3, respectively, with a complication rate of 26% (Strelzow et al., 2017). Baek et al. reported satisfactory results of monopolar prostheses for complex radial head fractures, with a mean MEPS of 88.7 ± 11.5 and a mean DASH score of 19.4 ± 7.8 (Baek et al., 2020). In the present study, the rates of complications and reoperation with uncemented monopolar protheses were 24% and 14%, respectively; the rates with cemented monopolar prostheses were 32% and 36%, respectively. The mean VAS score, MEPS, DASH score, and Broberg–Morrey score were 0.8–3.3, 70–91.5, 6.3–34, and 63–78.4, respectively. Bipolar radial head implants were invented to improve the junction and ROM of the elbow, and some experts suggest that their use leads to better elbow kinematics after surgery. Laun et al. reported good-to-excellent results of bipolar arthroplasty for radial head fractures at an average of 5.6 years of follow-up (Laun et al., 2019). Patients showed a mean DASH score of 18.6 and a mean MEPS of 90.3. Burkhart et al. also reported promising mid-to-long-term results with bipolar radial head prostheses. The mean MEPS reached approximately 90.8, and the mean DASH score reached approximately 9.8. However, Burkhart et al. reported degenerative changes in 71% and periarticular ossification after surgery in 76%, which were confirmed in the present study (Burkhart et al., 2010). Herein, the rates of complications and reoperation with uncemented bipolar protheses were 24% and 14%, respectively; the rates with cemented bipolar protheses were 32% and 36%, respectively. The mean VAS score, MEPS, DASH score, and Broberg–Morrey score were 0.2–1.4, 78.9–95, 8–23.9, and 86.5–86.6, respectively.

The efficacy of bipolar or monopolar prostheses is still controversial. Theoretically, bipolar designs reduce abrasion of the capitellar cartilage and stress at the implant-to-cement and cement-to-bone interfaces because of the free rotation between the stem and articular components. The radiocapitellar contact pressure may also decrease with bipolar designs compared with that with monopolar designs owing to the better alignment of the articular component onto the capitellum. Antoni et al. reported similar clinical and radiological results and complication and revision rates (Antoni et al., 2020). Hejink et al. reviewed 30 studies involving 727 patients and found that there was no significant difference in the ROM or clinical outcomes between bipolar and monopolar prostheses (Heijink et al., 2016). Mukka et al. conducted a mean follow-up of 6 years between two kinds of prostheses and found no significant difference in the QuickDASH score and ROM (Mukka et al., 2020). Van Riet et al. reviewed radial head prostheses revisions and observed a lower incidence of loosening with fixed-stem bipolar prostheses than with monopolar prostheses (Van Riet et al., 2010). In the survey by Antoni et al., the rate of ectopic ossification was higher in monopolar prostheses, which may be attributed to the longer follow-up in the monopolar prostheses group.

In our study, cemented monopolar prostheses yielded the highest reoperation rate, while uncemented monopolar prostheses yielded the lowest reoperation rate. Similarly, cemented bipolar prostheses had the highest complication rate, while uncemented bipolar prostheses had the lowest complication rate. Thus, the effect of cemented or uncemented fixation may be dominant in the outcome of RHA, with a minimal effect of bipolar or monopolar fixation. However, further biomechanical and clinical studies are required.

## Limitation

As with any systematic review or meta-analysis, our study has several limitations. First, because data interpretation depends on the quality of the information gathered, the validity of our study may be limited by the respective levels of evidence. Second, different prostheses differ in morphological design, which may have an impact on the results. Third, in the subgroup analysis, only three studies mentioned the rate of periprosthetic loosening in uncemented bipolar prostheses, while three studies mentioned the rate of overall complications in monopolar cemented prostheses, which would create a significant bias. Finally, we included only English literature; studies in other languages were not included, and the results may be affected by the fact that the prostheses used worldwide vary.

## Conclusion

Uncemented radial head prostheses have lower rates of reoperation and overall complications than cemented radial head prostheses. In particular, uncemented monopolar prostheses may yield the lowest rate of reoperation, while uncemented bipolar prostheses may yield the lowest rate of overall complications.

## Data Availability

The original contributions presented in the study are included in the article/supplementary material, further inquiries can be directed to the corresponding author.
